# The Contribution of Endothelial Dysfunction in Systemic Injury Subsequent to SARS-Cov-2 Infection

**DOI:** 10.3390/ijms21239309

**Published:** 2020-12-06

**Authors:** Jessica Maiuolo, Rocco Mollace, Micaela Gliozzi, Vincenzo Musolino, Cristina Carresi, Sara Paone, Miriam Scicchitano, Roberta Macrì, Saverio Nucera, Francesca Bosco, Federica Scarano, Maria Caterina Zito, Stefano Ruga, Annamaria Tavernese, Vincenzo Mollace

**Affiliations:** 1 Institute of Research for Food Safety & Health IRC-FSH, University Magna Graecia, 88100 Catanzaro, Italy; jessicamaiuolo@virgilio.it (J.M.); rocco.mollace@gmail.com (R.M.); micaela.gliozzi@gmail.com (M.G.); xabaras3@hotmail.com (V.M.); carresi@unicz.it (C.C.); sara.paone06@gmail.com (S.P.); miriam.scicchitano@hotmail.it (M.S.); robertamacri85@gmail.com (R.M.); saverio.nucera@hotmail.it (S.N.); boscofrancesca.bf@libero.it (F.B.); federicascar87@gmail.com (F.S.); mariacaterina.zito@studenti.unicz.it (M.C.Z.); rugast1@gmail.com (S.R.); an.tavernese@gmail.com (A.T.); 2 Nutramed S.c.a.r.l., Complesso Ninì Barbieri, Roccelletta di Borgia, 88021 Catanzaro, Italy; 3 Department of Medicine, Chair of Cardiology, University of Rome Tor Vergata, 00133 Roma, Italy; 4 IRCCS San Raffaele Pisana, 00163 Roma, Italy

**Keywords:** SARS-CoV-2, angiotensin-converting enzyme 2, endothelium dysfunction, thrombosis, vasculitis

## Abstract

SARS-CoV-2 (Severe Acute Respiratory Syndrome Coronavirus 2) infection is associated, alongside with lung infection and respiratory disease, to cardiovascular dysfunction that occurs at any stage of the disease. This includes ischemic heart disease, arrhythmias, and cardiomyopathies. The common pathophysiological link between SARS-CoV-2 infection and the cardiovascular events is represented by coagulation abnormalities and disruption of factors released by endothelial cells, which contribute in maintaining the blood vessels into an anti-thrombotic state. Thus, early alteration of the functionality of endothelial cells, which may be found soon after SARS-CoV-2 infection, seems to represent the major target of a SARS CoV-2 disease state and accounts for the systemic vascular dysfunction that leads to a detrimental effect in terms of hospitalization and death accompanying the disease. In particular, the molecular interaction of SARS-CoV-2 with the ACE2 receptor located in the endothelial cell surface, either at the pulmonary and systemic level, leads to early impairment of endothelial function, which, in turn, is followed by vascular inflammation and thrombosis of peripheral blood vessels. This highlights systemic hypoxia and further aggravates the vicious circle that compromises the development of the disease, leading to irreversible tissue damage and death of people with SARS CoV-2 infection. The review aims to assess some recent advances to define the crucial role of endothelial dysfunction in the pathogenesis of vascular complications accompanying SARS-CoV-2 infection. In particular, the molecular mechanisms associated with the interaction of SARS CoV-2 with the ACE2 receptor located on the endothelial cells are highlighted to support its role in compromising endothelial cell functionality. Finally, the consequences of endothelial dysfunction in enhancing pro-inflammatory and pro-thrombotic effects of SARS-CoV-2 infection are assessed in order to identify early therapeutic interventions able to reduce the impact of the disease in high-risk patients.

## 1. Introduction

Observational studies carried out in the development of coronavirus disease 2019 (Covid-19) pandemic revealed that the cardiovascular system represents one of the major targets of Severe Acute Respiratory Syndrome Coronavirus 2 (SARS-Cov-2) infection. In fact, it has been found that nearly 30% of SARS-CoV-2 patients undergo cardiac injury and that cardiovascular complications including acute cardiac injury, stroke, heart failure, arrhythmias, and cardiomyopathies may be detected at any stage of the infection [1]. The prevalence of cardiovascular co-morbidities, such as diabetes, hypertension, or coronary artery disease, is often associated with an unfavourable prognosis of SARS-CoV-2 infection [2]. Clinical studies in patients with SARS-Cov-2, both in China and in Italy, showed a higher mortality rate (CFR) in patients with cardiovascular co-morbidity than those without co-comorbidity [3,4,5]. Furthermore, a recent clinical study of 44,672 SARS-Cov-2-infected patients showed the CFR increased from 0.9% (in the absence of co-morbidity) to 10.5% in the presence of cardiovascular disorders to 7.3% in the presence of diabetes and to 6% in the presence of hypertension [6]. The pathophysiological mechanisms underlying the higher incidence of cardiovascular complications detected in patients undergoing severe SARS-Cov-2 disease are still to be better clarified. However, the common pathophysiological link between SARS-CoV-2 infection and the cardiovascular events leading to irreversible multi-organ dysfunction is represented by the disruption of factors that maintain the blood vessels into an anti-thrombotic state. In fact, it is possible to highlight an association between SARS-CoV-2 infection and increased risk of thromboembolism [7].

In general, thrombotic response involves two important aspects: (1) platelet activation and (2) a coagulation cascade [8].

(1) At the moment of platelet activation, arachidonic acid is converted to thromboxane A2, which is a potent pro-aggregatory and vasoconstrictive factor [9]. Then the platelets degranulate and finally undergo a conformational change assuming a starry shape, which is characteristic of their physical aggregation [10].
(2) The activation of the coagulation cascade determines, through several pathways, the formation of thrombin that splits the fibrinogen into fibrin. This fibrillar protein polymerizes to form a “mesh” together with platelets at the wound site.

It is important to point out that there is an interaction between the coagulation cascade and the activation of the platelets. In fact, thrombin can activate platelets but also conversely platelets themselves are able to catalyze the formation of thrombin [11].

During an inflammatory process that activates leukocytes, with the recruitment and translocation of other leukocytes as well as the release of cytokines and other inflammatory mediators, activation of the complement and attempt to eliminate agents have infected or attacked cells [12]. Platelets play an important role in the regulation of the inflammatory process. During acute inflammation, there is an increase in platelet-monocyte and platelet-neutrophil aggregates. In addition, platelet inhibition reduces cytokines such as Interleukin 6 (IL-6) and Tumor Necrosis Factor alpha (TNF-α) [13]. 

Multiple mechanisms have been suggested to approach this phenomenon. In particular, evidence exists that SARS-CoV-2 infection produces an aggressive and systemic pro-inflammatory response in which viral infection is associated with a consistent release of mediators of inflammation known as the so-called “cytokine storm” [14,15]. This leads to impressive damage of lung tissue, which becomes unable to ensure the due oxygen exchange in pulmonary tissue. Moreover, SARS-CoV-2 infection-induced hypoxia is accompanied by an enhanced tendency to develop thrombosis of blood micro-vessels via an increased blood viscosity [16]. This is also related to the general status of hospitalized patients, who are more than 60 years old, bedridden for a long time, and subjected to invasive treatments. These are risk factors to develop hyper-coagulation or thrombosis [17].

The common pathophysiological feature, which can be found in patients with irreversible cardio-respiratory complications occurring in SARS-Cov-2 disease is represented by an imbalanced anti-coagulant activity of vascular endothelial cells, which is also called “sepsis-induced coagulopathy” (SIC). This is an effect that leads to an increased risk of thrombotic events [18]. For this reason, although the correlation between SARS-CoV-2 infection and coagulation disorders is still unclear, a tight monitoring of coagulation biomarkers was suggested in high-risk patients [19].

SARS-CoV-2 infection associated coagulopathies are characterized by high levels of D-dimer (a fibrin degradation product detectable in the blood in case of fibrinolysis), fibrinogen, prothrombin time (PT), and thrombocytopenia [20]. Furthermore, this condition is accompanied by a micro-thrombosis, systemic inflammatory response, and impairment of vascular reactivity [21]. 

Finally, a systemic impairment of endothelial function seems to occur in the majority of patients undergoing SARS-CoV-2 infection, thereby, playing a crucial role in the SARS-CoV-2-linked sepsis-related coagulopathy [22]. In particular, emerging evidence suggests that SARS-CoV-2 could damage the endothelial barriers and this event could contribute to the severe and systemic condition generated by pandemic infection. 

The present review aims to assess some of the most recent evidence identifying the major targets of SARS-CoV-2 underlying endothelial dysfunction in SARS-Cov-2 disease state. In addition, evidence for the systemic and multi-organ endothelial impairment occurring in SARS-Cov-2 infection will be discussed with the aim to detect potential therapeutic targets to be useful in the course of the disease.

## 2. Major Molecular Targets of SARS-CoV-2

Coronaviruses (CoVs) (Nidovirales order, Coronaviridae family, Coronaviridae subfamily) are a comprehensive family of viruses with different phenotypic and genotypic characteristics [23]. There are six known human endemic CoVs, which are called, respectively, HCoV-229E, HCoV-NL63, HCoV-OC43, and HCoV-HKU1. In addition, two further CoVs have been detected and characterised over the last 20 years. They are: Severe Acute Respiratory Syndrome (SARS)-CoV (identified in 2003) and the Middle East Respiratory Syndrome (MERS)-CoV (identified in 2013) [24]. SARS-CoV and MERS-CoV belong to the β-CoV genus and both have caused very severe lung dysfunction [25]. In December 2019, the first cases of a new coronavirus called SARS-CoV-2 were reported and SARS-CoV-2 has been identified to be responsible of the ongoing pandemic infection worldwide [26]. The SARS-CoV-2 genome consists of a single positive-polarity RNA strand (+ssRNA) with a number of bases ranging from 27 to 33 kb. The organization of the SARS-CoV-2 genome is polycistronic and RNA is translated to produce a single polyprotein that is later broken down by proteases to obtain 16 non-structural proteins of the virus, making up the viral replication-transcription complex. Moreover, the region near the 3′ end of the viral genome carries information for the main four viral structural proteins: spike (S), membrane (M), envelope (E), and nucleocapsid (N). The homotrimer of the S proteins generates the spikes on the external part of the virus, which are involved in the virus coupling to the receptors of the host cell. It is important to note that SARS-Cov-2 has an important mutation, compared to previous CoVs, that affects the Spike protein, called D614G. The D614G mutation seems to make up to eight times more effective S protein translation [27] and has been shown to increase the infectious capacity of s in human lung epithelial cells [28]. In vitro experiments have shown that there is a minimal difference in ACE2 receptor binding between S variants. However, the form G614 is more resistant to proteolytic cleavage and this may indicate a potential mechanism to justify increased S protein translation [29]. These findings determine important implications in the effectiveness of any Spike-based vaccines [30].

**Figure 1:**
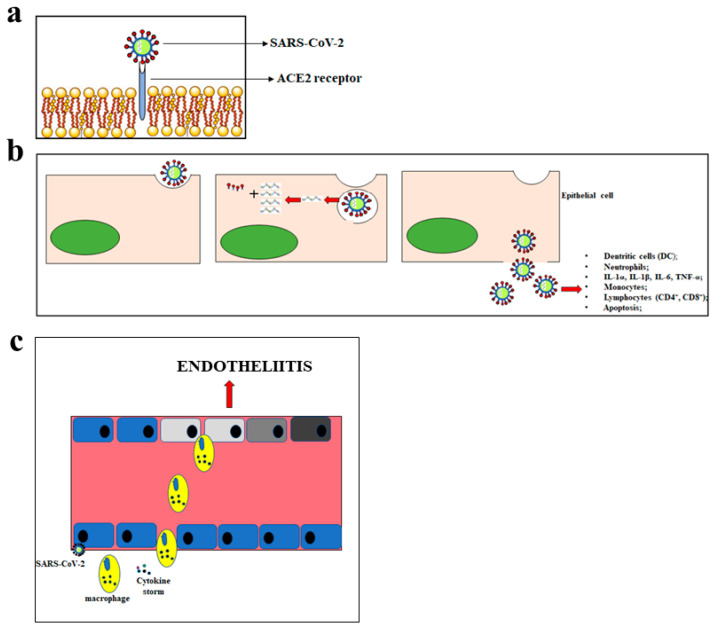
Main consequences of the SARS-CoV-2 penetration. In panel (**a**), the mechanism of SARS-CoV-2 penetration into the cells is represented. Panel (**b**) shows the massive inflammatory response following the cellular introduction of SARS-CoV-2. In panel (**c**), inflammation of the endothelium and the endotheliitis process are shown.

M protein binds to the nucleocapsid and favours the curvature of the host cell membrane. E protein takes part both in the assembly and in the release of the virus. Finally, protein N deals with genome packaging in virions [31]. The first CoV replication step is the ability to penetrate the target cells. To this end, the efficacy of the binding between the S glycoprotein of CoVs and the protein receptor on the cell surface is crucial [32]. SARS-CoV and SARS-CoV-2 use the same receptor, which is angiotensin-converting enzyme 2 (ACE2). Conversely, MERS-CoV uses the Dipeptidyl Peptidase 4 (DPP4) receptor [33]. The binding of the virus spikes with the host receptor that leads to the binding of their membranes, the penetration of the virus into the host by endocytosis, and the beginning of the life cycle of the virus. The virus releases its viral genome, synthesizes viral structural proteins and the genome, and assembles mature virions released via an exocytosis mechanism [34]. The SARS-CoV-2 infection is characterised by various symptoms and may be even lethal. The main symptoms include fever, cough, short breath, pneumonia, fatigue, severe respiratory distress, hepatic and gastrointestinal disorders, lymphopathy, and neurological diseases. The symptoms manifest themselves about 14 days after the exposure to the virus [35]. The main mode of transmission of SARS-CoV-2 is through respiratory droplets, leading to person-to-person spread with each infected individual, on average, causing 2–3 new infections [36]. The main cellular consequences of SARS-CoV-2 penetration are shown in Figure 1.

## 3. ACE2 Receptor Characterization

The renin-angiotensin system (RAS) performs several body functions at the systemic and local level [37]. The systemic RAS is responsible for maintaining proper blood pressure and electrolyte homeostasis whereas local RAS regulates many functions of the heart, kidney, and lung. For this reason, RAS is the most important regulator of the cardiovascular and renal function and this balanced regulation is guaranteed by proteases that hydrolyze bioactive circulating peptides. 

The glycoprotein angiotensinogen is cleaved by the enzyme renin, secreted into the circulation in response to numerous stimuli, to produce the decapeptide angiotensin I (Ang I). Ang I can be cleaved to the octapeptide angiotensin II (Ang II) by the angiotensin-converting enzyme (ACE). Ang II is the main bioactive component within RAS and can mediate vasoconstrictive or vasodilatory effects depending on the receptor to which it binds. Ang II can further be processed to produce the vaso-dilator heptapeptide Ang 1–7 by angiotensin-converting enzyme 2 (ACE2) [38]. In particular, the peptidase domain of ACE2 cleaves Ang II at the level of amino acid phenylalanine. ACE2 can also cleave Ang I, at the level of amino acid leucine, creating the biological inactive peptide Ang1–9. ACE2 shows a greater bond affinity for Ang II than Ang I [39]. To date, cumulative results suggest a deleterious role of ACE and a protective role of ACE2 due to their attitude to produce and degrade Ang II, respectively [40]. An abnormal activation of RAS leads to diseases such as heart failure, myocardial infarction, and renal diseases [41]. 

The human ACE gene is located on chromosome 17 and encodes a type-I transmembrane glycoprotein anchored to the plasma membrane and consisting of two homologous domains. The human ACE2 gene is located on the X chromosome and encodes a type I transmembrane glycoprotein. ACE2′s amino-terminal and carboxy-terminal domains show 41.8% and 48% sequence identity with the respective ACE domains [42]. Scientific studies of literature have shown that gene deletion of ACE2 resulted in impaired cardiac contractility and this heart dysfunction was attributed to increased Ang II levels [43]. Both ACE and ACE2 are zinc metallopeptidase angiotensin-converting enzymes and are characterised by a similar intermembrane structure [44]. Nevertheless, ACE1 and ACE2 show many differences.

- Different catalytic activity: ACE is a dipeptidase that hydrolyses bound pairs of amino acids cleaving the C-terminal dipeptide from Ang I to form the octapeptide Ang II. ACE2 is a carboxypeptidase capable of breaking peptide bonds between amino acids at the level of terminal C residue and removing the residue from the decapeptide Ang I to form angiotensin-1–9.
- Several substrates and bond specificity: in particular, ACE binds and cleaves Ang I, Ang 1–9, and many bioactive peptides. In contrast, ACE2 cleaves Ang I, Ang II, apelin-13, and apelin-36 [45].
- A different expression in different tissues of the organism has been shown: ACE is more ubiquitous. In fact, this enzyme is expressed in the heart, lung, kidney, colon, small intestine, ovary, testis, prostate, liver, skeletal muscle, pancreas, and thyroid. In contrast, the expression of ACE2 is more specific and is limited in the heart, kidney, endothelial cells, and microvasculature [46,47].
- Inhibitor specificity: The ACE inhibitors act by blocking of the conversion of angiotensin I to angiotensin II and inhibiting the most important step in the RAS pathway. For this reason, they are widely used as a class of anti-hypertensive drugs. The ACE inhibitors have been linked to cases of hepatotoxicity. ACE2 cannot be inhibited to ACE inhibitors [48].

Since ACE2 decreases the levels of the vasoconstrictor Ang II and produces the vasodilator Ang- (1–7), it may protect against cardiovascular and renal disease [49]. ACE2 has two characteristics that allows its characterization:

1. The catalyzed peptides are preferably hydrolysed on the proline residues to the C-terminal group [50].
2. Its enzymatic activity is regulated by chloride ions. The bond with the chloride induces conformational changes of the active site of the enzyme, which is responsible for modulation of the reactions [51].

It is important to note that ACE2 performs important biological functions alongside its peptidase role known in the RAS system [52]. For example, it acts as a carrier of amino acids. In particular, evidence exists that the proteins taken with the diet, are digested by hydrolysis reactions carried out by the proteases of the stomach and pancreas while tripeptides, bipeptides, and neutral amino acids are absorbed in the intestine by the major small intestine luminal transporter B^0^AT1 [53]. Intestinal B^0^AT1 expression and function depends on the concomitant presence of the accessory protein ACE2 [54]. The deficiency of ACE2 compromises amino acid absorption in the mouse [55]. Pharmacological inhibition of B^0^AT1 reduces protein absorption and increases the elimination of amino acids through the urine [56].

### Molecular Mechanisms of ACE-2-Covid 19 Interaction

Another biological function performed by the transmembrane domain of ACE2 was recognized in 2003 during the epidemics of Coronavirus SARS. In fact, ACE2 was identified as a functional receptor for this pathogenic agent. Structural analyses have been conducted and it has been highlighted that the SARS-Cov spike protein interacts with subdomain I of ACE2 but does not affect the subdomain II nor occludes the peptidase active site [57]. The demonstration of this hypothesis has been provided by experiments in which cells expressing ACE2 catalytic inactive mutants were still permissive for SARS-CoV infection. These results have definitively excluded the involvement of the ACE2 peptidase site in the SARS-CoV penetration in the host cell [58]. After the link between SARS-CoV and ACE2 has been made, the outer portion of ACE2 is split and released while the intramembrane portion is internalized and used to facilitate the fusion of the virus to the host cell [59,60,61]. As already mentioned, similarly to SARS-CoV, SARS-CoV-2 uses ACE2 as a receptor to penetrate the host cell. SARS-CoV-2 consists of a homo-trimeric spike protein in which these ends protrude from the viral surface and facilitate the attachment and adhesion of viruses to human target cells [62]. The protein Spike is composed of two subunits known as S1 and S2. S1consists of four domains named S1A, S1B, S1C, and S1D, dealing with receptor association and stabilization of this bond. In particular, S1A engages host sialic acids and S1B recognizes host transmembrane proteins. The S2 domain is responsible for membrane fusion [63]. Some experimental structural and biophysical tests have shown that S proteins of SARS-CoV-2 bind to the ACE2 receptor of the host cell with an affinity 10–20 times higher than the one reached by other known members of the SARS-CoV family [64]. 

The characteristics of SARS-CoV-2 to enter the host cell are sufficiently known. To date, much interest has arisen on the correlation between the ACE2 receptor and the onset of vascular dysfunction related to SARS-CoV-2 progression. An interesting hypothesis that is currently being evaluated is the possibility that the entry of the SARS-CoV-2, by the ACE2 receptor, favors a down-regulation of the receptor expression, induces vascular endothelial dysfunction, and, thereby, activates a pro-thrombotic cascade. Since ACE2 is an analogue of ACE, which is a key regulator of the RAS system regulating hemodynamic homeostasis in the body, a balanced ACE/ACE2 ratio is fundamental for maintenance of endothelial integrity in vessels [65]. On the contrary, dysregulation of this ratio could be associated with vascular thrombosis [66,67]. This hypothesis is confirmed by in vitro studies carried out on lung tissues infected with SARS-CoV. It has been demonstrated that the link between the virus and ACE2 regulates the expression of the receptor, induces an ACE/ACE2 imbalance, and promotes prothrombotic cascades inside the vessels [64]. A low ACE/ACE2 ratio is responsible for a greater degradation of Ang II to form Ang 1–7, which plays an anti-thrombotic action preventing the activation of the pro-thrombotic cascade in the vascular endothelium [68]. In contrast, a high ACE/ACE2 ratio will cause an increase in Ang II, the binding of the latter to AT1 receptors, and the onset of vasoconstriction, inflammation, and thrombosis [69,70]. Some scientific studies have shown that *ACE2* knockout mice develop a vascular endothelial dysfunction, responsible for pro-inflammatory processes [71], platelet aggregation, and thrombosis [69]. At the same time, ACE2 activation has been shown to protect against vascular thrombosis [70]. ACE inhibitors and angiotensin receptor blockers have been used as a treatment in patients with congestive heart failure. Until now, there has been great uncertainty about the effectiveness of using these drugs in conjunction with SARS-CoV-2 infection. A recent study, conducted in England on 1205 patients, aged 20–99, found that the ACE inhibitor drug was associated with a reduced risk of contracting SARS-CoV-2 infection [71]. Further studies, however, would be desirable.

## 4. Endothelium Dysfunction and SARS-CoV-2

The endothelium can be considered a real organ that constitutes the internal cellular lining of the blood vessels and understands a selectively permeable barrier composed of a layer of endothelial cells. These cells do not perform a passive role. In contrast, they regulate very important physiological functions such as maintaining the homeostatic balance, controlling the vasomotor tone, guaranteeing the correct permeability, and managing the reactions of innate immunity [71]. Endothelial permeability allows the physiological transport of a few necessary molecules. Normally, endothelial cells can be traversed by water and small solutes while the larger solutes pass through trans-endothelial channels or transcytosis. Correct permeability of the endothelium is regulated through junction proteins whose function is to maintain closely adjacent endothelial cells. In this way, it is impossible to pass unwanted molecules or cells [72,73]. Junction proteins are made up of tight junctions and adherens junctions. The loss or reduction of these proteins’ expression leads to disassembly and endothelial dysfunction [74]. The endothelium also constitutes the first defensive barrier against foreign invasion that include mechanical and chemical stimuli. Generally, the integrity of the endothelial barriers depends on the delicate balance between the intracellular contraction and the cell-cell and cell-matrix adhesiveness. Specifically, the cytoskeleton proteins play a key role to ensure the cell adhesions and junctions [75,76]. At a pulmonary level, the respiratory activity takes place in the alveolus-capillary unit that consists of an alveolus supplied by blood capillaries. In particular, the alveolus is covered by an alveolar epithelium separated from the capillary by an intermediate space. Eventually, the capillary is surrounded and protected, both internally and externally, by a layer of endothelial cells. Therefore, the pulmonary endothelium acts as a semi-permeable barrier between the blood and the intermediate space. The pulmonary epithelial and endothelial cells are kept close by many tight junction proteins [75]. From the structural point of view, the cells grown together in this unit are enclosed in a small space with an average thickness of about 0.5 μm [76]. On their luminal side, the endothelial cells are covered by a glycocalyx consisting of a network of proteoglycans and glycoproteins involved in cell-cell signaling processes. Moreover, pericyte cells are observed, which adhere to endothelial cells and act as mediators in the various microvascular processes, such as the endothelial cell proliferation and angiogenesis. Pulmonary vascularization is extensive in human beings and would cover a total surface of 90 m^2^. The pulmonary endothelium is one of the main components of the alveolus-capillary unit and the safeguard of its integrity is extremely important. In this respiratory system, the endothelium may be subjected to structural changes triggered by both mechanical factors and the invasion of pathogens. Any damage to the endothelial cells leads to the interruption of the barrier, an increased permeability, and an inflow of molecules that generate inflammatory responses [77]. Pulmonary endothelium is able to generate bioactive molecules and/or to use compounds present in the cell to reduce the effects of toxic stimuli and restore its conditions. If the damage is particularly extensive, the endothelium cannot tackle it and its permeability undergoes some alterations. The pulmonary endothelial barrier is completely destroyed in the case of chronic lung damages. A typical example of chronic inflammation of the alveolus-capillary unit is represented by virus infections. This process occurs, for example, when the organism comes into contact with the common flu virus [78] In fact, the virus infection impairs both the alveolar epithelium and the pulmonary endothelium. The alveolar epithelium is damaged as a result of the viral entry and the virus replication capacity, which can determine the severity of the infection. The impairment of the pulmonary endothelium is caused by the host’s adaptive immune response to the virus [79]. Any complication of the seasonal flu, which can lead to severe changes in some patients, cannot be justified exclusively by the destruction and/or apoptotic death of the pulmonary epithelium cells. It is worth considering the effect of the virus on the other side of the alveolar-capillary membrane, i.e., the pulmonary endothelium. The main change, at the endothelial level, is due to the higher permeability with the internalization of the immune cells (lymphocytes, monocytes, and neutrophils) as well as the internalization of their inflammatory cytokines. This scenario results in further systemic aggravation [80]. In particular, the pro-inflammatory cytokines produced by leukocytes, the pulmonary epithelium, and the pulmonary endothelium further impair the pulmonary permeability [81,82]. In short, the flu virus infection is associated with an infiltration of white blood cells in the lungs. Neutrophils, in particular, release cytokines, reactive oxygen species, elastases, and nucleic acids that contribute to the destruction of the endothelial barrier. Moreover, cytokines and other inflammatory mediators impair the endothelial cell-cell junctions with the following formation of inter-cellular interstices, causing the loss of vascular fluid in the intermediate space, resulting in oedemas. The mechanisms underlying the junctional destruction include the reduction of Cadherin 5, type 2 (VE cadherin), which is a protein that ensures the cohesion of the endothelial cells, and the cytoskeletal rearrangement of the actin filaments [83].

Considering the previous assumptions concerning the endothelium, it would be interesting to imagine a model of the impairment of the alveolus-lung unit resulting from the virus infection caused by SARS-CoV-2. In fact, the cells of the pulmonary endothelium express the ACE2 receptor [73], and it would be argued that the virus, after having attacked and destroyed the pulmonary epithelium, could transmigrate to the underlying endothelium and penetrate the cells [84]. Thus, through the mechanisms already explained, SARS-CoV-2 could damage the endothelium and contribute to the severe and systemic condition generated by this pandemic infection.

A very interesting recent scientific work has shown the involvement of endothelial cells across vascular beds of different organs in a series of patients with SARS-CoV-2. The results obtained are related to people with SARS-CoV-2, with previous diseases, that, following the infection, died. A subsequent autopsy was carried out. Post-mortem analysis revealed the presence of viral elements within the endothelium and an accumulation of inflammatory cells that led to the death of endothelial cells. In addition, histopathological analysis showed that SARS-Cov-2 infection led to the induction of endothelitis in different organs. Finally, apoptosis and pyroptosis of endothelial cells were also highlighted [85].

Under normal conditions, the endothelium is anticoagulated by a series of natural anticoagulant systems. In case of damaged endothelium, there is a cellular alteration which, in many cases, leads to an inflammatory state known as “endotheliopathy.” In this altered condition, a dysfunction in the microcirculation occurs [86]. Endothelitis and SARS-CoV-2 inclusions were found in the vascular endothelial cells of patients who died as a result of infection and underwent autopsy. These findings have indicated alteration of endothelial cells as a central feature of the pathophysiology of SARS-CoV-2 during the inflammatory phase of the disease [87]. The concrete proof of this deduction was provided by a biochemical measurement of specific markers of endotheliopathy, haemostatic factors, and platelet activation, which were always altered in the coagulopathy associated with SARS-CoV-2 infection [88]. The Von Willebrand factor (vWF) is a large multimeric glycoprotein that performs two critical functions in primary hemostasis. It acts as a bridge molecule in vascular lesion sites for normal platelet adhesion and, under high shear conditions, promotes platelet aggregation. Factor VIII (FVIII) is an essential blood-clotting protein, also known as an anti-hemophilic factor (AHF). vWF together with AHF are common in people with SARS-CoV-2 and their levels were found to be much higher than for unaffected patients [89].

**Figure 2:**
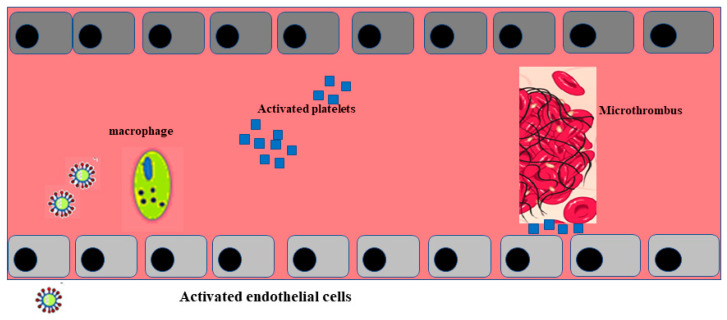
Activation of endothelial cells and formation of a microthrombus. The sequence of endothelial inflammation, activation of endothelial cells, and, as a result, the formation of a microthrombus are shown.

Post mortem examination of 21 people diagnosed with SARS-CoV-2 has shown that the primary cause of death was respiratory failure with massive capillary congestion and severe changes of rheological properties in capillaries. Nevertheless, subsequent findings have highlighted pulmonary embolisms (in four patients), alveolar haemorrhage (in three patients), thrombotic microangiopathy (in three patients), and vasculitis (in one patient). The observation of all patients also highlighted damage of the endothelial in the kidneys and intestines, suggesting vascular dysfunction in disease progression [90]. Another important study, carried out with an autoptic examination of 26 people who died as a consequence of SARS-CoV-2 infection, demonstrated kidney injuries with glomerular and vascular changes, occlusion of the microvascular lumens, and numerous erythrocytes as a consequence of endothelial damage [91]. The damage to the endothelium and the formation of a microthrombus, caused by SARS-CoV-2 infection, are represented in Figure 2.

## 5. SARS-CoV-2 Related Vasculitis

Vasculitis is a specific inflammation of the blood wall and can affect not only the skin but any organs of the body. They are classified according to the size (small, medium, or large) of the involved vessel. Most vasculitic lesions are related by immunopathogenic mechanisms classified in (1) allergic vasculitis, (2) antibody-mediated vasculitis, (3) IC-mediated vasculitis, and (4) T cell–mediated hypersensitivity vasculitis [92]. It has recently been highlighted that there is a direct correlation between SARS-CoV-2 infection and dermatological manifestations such as erythema, rash, urticarial lesions, and varicella-like vesicles common to other viral infections. The described lesions appear in a colour ranging from red to purple and can evolve into vesicles similar to chilblains. Moreover, the cutaneous vasculitis–like manifestations are considered to be a pathognomonic sign of SARS-CoV-2 infection [93]. Some research groups have reported that the co-occurring presence of skin manifestations and SARS-CoV-2 symptomatology rules out a reaction to drugs since these patients reported having no recent history of drug intake before the viral infection [94].

The first study, conducted in Italy (Lecco hospital, Lombardy, Italy) on 88 patients affected with SARS-CoV-2 infection, showed that n = 18 (20.4%) developed cutaneous involvement. Among these, eight patients experienced symptoms at the onset of the disease, while 10 patients experienced symptoms after hospitalization. Cutaneous manifestations were the erythematous rash (14 patients), widespread urticaria (three patients), and chickenpox-like vesicles (one patient). The most affected region of the body was the trunk and usually lesions healed in a few days, showing that they are benign [95]. Moreover, patients with dermatological symptomatology have not always been hospitalized since they did not present, at that moment, the typical symptomatology of SARS-CoV-2 infection. This suggested that the patients affected with SARS-CoV-2 might initially present with a skin rash that can be exchanged as another common disease and may subsequently develop canonical SARS-CoV-2 symptomatology. Therefore, dermatological manifestations of SARS-CoV-2 could be considered as a prognostic reference. The number of reports is quickly growing in Italy and in many European countries and seems to overlap the SARS-CoV-2 pandemic propagation [80]. A recent study reported that the cutaneous lesions are preferably shown in asymptomatic or mildly symptomatic pediatric patients with prevalent involvement in the foot and hand [96]. 

A prospective study conducted in Spain reported that 375 cases of patients showed skin lesions associated with SARS-CoV-2 infection. This study also indicated a temporal relationship between skin lesions and other systemic symptoms. In particular, skin lesions appeared in the early course of the disease and, at a later time, appeared to have other systemic symptoms [97].

This phenomenon could be due to endothelial cell dysfunction that induces a cytokine storm, recruits macrophages, and causes inflammatory reactions, similar to those of vasculitis [98]. Currently there are few papers about the dermatological manifestations of SARS-CoV-2 and we need more knowledge and experience to understand this probable correlation.

## 6. Therapeutic Interventions in Endothelial Dysfunction and in Sars-Cov-2 Systemic Damage

The main mechanisms involved in endothelial dysfunction include increased oxidative stress, down-regulation of endothelial nitric oxide synthase levels, endoplasmic reticulum stress, and altered expression of a vascular endothelial growth factor that induces vascular complications such as microvascular disease and coronary artery disease [99,100]. When the endothelium is damaged, the same endothelial barrier cells can reduce damage through three mechanisms.

(1) Activation of GTPase enzymes triggering a self-repair process [101]. Following the dangerous stimuli, that alter the integrity of the endothelium, small enzymes with GTP-ases functions are activated, which, through their internal cross-talk, promote the hydrolysis of the GTP. These GTPases enzymes control the integrity of the endothelial barrier by stimulating the genesis of actin paracellular fibers and sealing some junctional gaps [101].
(2) Recovery of molecules that combat barrier breaking [102]. Some molecules can counteract any endothelial barrier failure by preventing its rupture. They include cAMP and cAMP derivatives that increase endothelial cell functions through the protein kinase A (PKA) pathway. In particular, caderin expression, which promotes the formation of paracellular adhering junctions in endothelial cells, is up-regulated [103]. Another important molecule involved in this process is phospholipid oxidized 1-palmitoyl-2-arachidonoyl-sn-glycer-3-phosphorylcholine (Oxpapc). Oxpapc limits the effects of proinflammatory agents, such as thrombin, participating in remodeling of endothelial cytoskeleton and favoring maintenance of the endothelial cytoskeleton by facilitating the assembly of tight and adherent junctions [104].
(3) Secretion of growth factors. Growth factors improve the integrity of the endothelial barrier or reduce its permeability through the redistribution of actin filaments [105,106].

If the damage is particularly extensive, the endothelium cannot tackle it, and its permeability undergoes some alterations.

Many studies confirmed the protective effects of polyphenol and flavonoids’ antioxidants against many pathological pathologies including neurodegenerative [107], vascular [108], and cardiovascular diseases [109]. Furthermore, numerous scientific investigations have highlighted the protective role of polyphenols against endothelial and microvascular dysfunctions [110,111].

Some strategies are known to improve endothelial function. Among these, we will develop the use of colchicine. Recently, it was suggested that colchicine may be effective in SARS-CoV-2 infection and reduce the inflammatory cytokine storm during infection [112]. Colchicine is a lipid-soluble alkaloid extracted from plants of the genus Colchicum. It is a drug used to treat gout attacks through its anti-inflammatory properties, making it useful for several inflammatory conditions. Colchicine binds preferentially to free tubulin dimers by interrupting the further polymerization of the cellular microtubules. Impaired microtubular assembling leads to inhibition of vesicular transport, of cell migration and division, of phagocytosis, and of a cytokine secretory function of leukocytes [103]. Experimental studies have shown in a rat model of hyperlipidemia, that pharmacological treatment with colchicine could improve endothelial function through its anti-inflammatory effect [113]. In particular, colchicine’s activity in vasculitis is related to its properties for reducing cell adhesion molecules and decreasing the adhesion and migration of neutrophils. In a study conducted by Callen et al., 13 patients with chronic cutaneous vasculitis were treated with 0.6 mg colchicine twice daily. They reported that 12 patients responded within 10 days of starting therapy and nine of these achieved complete remission [113].

Among the pharmacological solutions that improve the SARS-CoV-2 condition is heparin. This molecule also acts by protecting against endothelial dysfunction.

Coagulopathy in SARS-CoV-2 infection, as already reported, has been shown to be associated with high mortality [114]. Treatment with heparin has shown interesting results [115]. Heparin interacts with the coagulation system and is widely used as an anticoagulant drug. Patients suffering from abnormal activation of the coagulation system and microthrombi formation need an anti-coagulant regulation. Several publications have shown that the main mechanisms, used by anti-coagulant drugs, involved inhibition of neutrophil chemotaxis, of leukocyte migration, and seizure of complement peptide factor C5a [116]. It has recently been demonstrated, both in vitro and in vivo, that heparin can protect the vascular endothelium stabilizing cytoskeletal microtubules and downregulating the nuclear factor-κB signaling pathway [114]. SARS-CoV-2 patients may have elevated D-dimer levels as a consequence of the massive inflammatory response caused by the infection. In fact, the immuno-thrombosis model shows a bi-directional relationship between the immune system and the generation of thrombin. The heparin could turn off and/or reduce the inflammatory response [115]. An interesting study has highlighted that people with SARS-Cov-2 infection, who had simultaneously high levels of D-dimer, had reduced the mortality rate when treated with heparin (32.8% against 52.4%) [116].

## 7. Conclusions

Similarities between SARS-Cov and SARS-CoV-2 are not only related to symptoms developed during infection or to the receptor mechanism of viral penetration. In fact, a common feature to both infections is to develop vascular thrombosis [117,118]. Abnormalities in the coagulation response, observed in SARS-CoV-2 infection, are not directly linked to an intrinsic characteristic of the virus but to its ability to trigger an inflammatory cascade [119]. Data reported directly from China, showed that 6% of 99 hospitalized patients affected by SARS-CoV-2 had a high prothrombin time along with a 36% of elevated D-dimer and increased biomarkers of inflammation [120]. Following a viral infection, a physiological inflammatory response is activated, which involves an alteration in the coagulation process. These events are involved in a process known as “thrombo-inflammation” or “immuno-thrombosis” [121,122].

In most patients who died of severe SARS-CoV-2 infection and who were autopsied, widespread thrombosis, and micro-vascularization were reported. Since thrombosis has also been observed in patients undergoing anticoagulant therapy, it is likely to believe that clotting disorders may be a characterizing factor of this infection [123].

According to this result, endothelial dysfunction represents a key mechanism that leads to early impairment of endogenous anti-inflammatory/anti-thrombotic responses of the vascular wall able to counteract systemic tissue damage subsequent to SARS CoV-2 infection. Moreover, the tendency to develop thrombotic vascular events associated with a SARS CoV-2 disease state must be taken into account when approaching people with SARS-CoV-2 even those who are asymptomatic. This should be crucial in preventing and treating SARS CoV-2–related systemic damage to reduce its impact in terms of hospitalization and death, which remains an unresolved issue in the course of the disease.

## References

[1] Akhmerov A., Marban E. (2020). COVID-19 and the heart. Circ. Res..

[2] Li B., Yang J., Zhao F., Zhi L., Wang X., Liu L., Bi Z., Zhao Y. (2020). Prevalence and impact of cardiovascular metabolic diseases on COVID-19 in China. Clin. Res. Cardiol..

[3] Wu Z., McGoogan J.M. (2020). Characteristics of and important lessonsfrom the coronavirus disease 2019 (COVID-19) outbreak in China: Summary of a report of 72 314 cases from the Chinese Center for Disease Control and Prevention. JAMA.

[4] Guzik T.J., Mohiddin S.A., Dimarco A., Patel V., Savvatis K., Marelli-Berg F.M., Madhur M.S., Tomaszewski M., Maffia P., D’Acquisto F. (2020). COVID-19 and the cardiovascular system: Implications for risk assessment, diagnosis, and treatment options. Cardiovasc. Res..

[5] Grasselli G., Zangrillo A., Zanella A., Antonelli M., Cabrini L., Castelli A., Cereda D., Coluccello A., Foti G., Fumagalli R. (2020). Baseline characteristics and outcomes of 1591 patients infected With SARS-CoV-2 admitted to ICUs of the Lombardy Region, Italy. JAMA.

[6] Kunal S., Gupta K., Sharma S.M., Pathak V., Mittal S., Tarke C. (2020). Cardiovascular system and COVID-19: Perspectives from a developing country. Monaldi Arch. Chest Dis..

[7] Al-Ani F., Chehade S., Lazo-Langner A. (2020). Thrombosis risk associated with COVID-19 infection. A scoping review. Thromb. Res..

[8] Gąsecka A., Borovac J.A., Guerreiro R.A., Giustozzi M., Parker W., Caldeira D., Chiva-Blanch G. (2020). Thrombotic Complications in Patients with COVID-19: Pathophysiological Mechanisms, Diagnosis, and Treatment. Cardiovasc. Drugs Ther..

[9] Parker W., Orme R.C., Hanson J., Stokes H.M., Bridge C.M., Shaw P.A., Sumaya W., Thorneycroft K., Petrucci G., Porro B. (2019). Very-low-dose twice-daily aspirin maintains platelet inhibition and improves haemostasis during dual-antiplatelet therapy for acute coronary syndrome. Platelets.

[10] Paul B.Z., Jin J., Kunapuli S.P. (1999). Molecular mechanism of thromboxane A(2)-induced platelet aggregation. Essential role for p2t(ac) and alpha(2a) receptors. J. Biol. Chem..

[11] Briedé J.J., Tans G., Willems G.M., Hemker H.C., Lindhout T. (2001). Regulation of Platelet Factor Va-dependent Thrombin Generation by Activated Protein C at the Surface of Collagen-adherent Platelets. J. Biol. Chem..

[12] Zhang Y., Liang C. (2016). Innate recognition of microbial-derived signals in immunity and inflammation. Sci. China Life Sci..

[13] Guo T., Fan Y., Chen M., Wu X., Zhang L., He T., Wang H., Wan J., Wang X., Lu Z. (2020). Cardiovascular Implications of Fatal Outcomes of Patients With Coronavirus Disease 2019 (COVID-19). JAMA Cardiol..

[14] Alijotas-Reig J., Esteve-Valverde E., Belizna C., Selva-O’Callaghan A., Pardos-Gea J., Quintana A., Mekinian A., Anunciacion-Llunell A., Miró-Mur F. (2020). Immunomodulatory therapy for the management of severe COVID-19. Beyond the anti-viral therapy: A comprehensive review. Autoimmun. Rev..

[15] Wong J.P., Viswanathan S., Wang M., Sun L.Q., Clark G.C., D’Elia R. (2017). Current and future developments in the treatment of virus-induced hypercytokinemia. Future Med. Chem..

[16] Nunes Kochi A., Tagliari A.P., Forleo G.B., Fassini G.M., Tondo C. (2020). Cardiac and arrhythmic complications in patients with COVID 19. J. Cardiovasc. Electrophysiol..

[17] Klok F.A., Kruip M.J.H.A., van der Meer N.J.M., Arbous M.S., Gommers D., Kant K.M., Kaptein F.H.J., van Paassen J., Stals M.A.M., Huisman M.V. (2020). Confirmation of the high cumulative incidence of thrombotic complications in critically ill ICU patients with COVID-19: An updated analysis. Thromb. Res..

[18] Iba T., Levy J.H., Warkentin T.E., Thachil J., van der Poll T., Levi M. (2019). Diagnosis and management of sepsis-induced coagulopathy and disseminated intravascular coagulation. J. Thromb. Haemost..

[19] Casini A., Fontana P., Glauser F., Robert-Ebadi H., Righini M., Blondon M. (2020). Venous thrombotic risk related to SARS-CoV-2: Prevalence, recommendations and perspectives. Rev. Med. Suisse..

[20] Tang N., Bai H., Chen X., Gong J., Li D., Sun Z. (2020). Anticoagulant treatment is associated with decreased mortality in severe coronavirus disease 2019 patients with coagulopathy. J. Thromb. Haemost..

[21] Hess D.C., Eldahshan W., Rutkowski E. (2020). COVID-19-Related Stroke. Transl. Stroke Res..

[22] Gavriilaki E., Brodsky R.A. (2020). Severe COVID-19 infection and thrombotic microangiopathy: Success doesn’t come easily. Br. J. Haematol..

[23] Rothan H.R., Byrareddy S.N. (2020). The epidemiology and pathogenesis of coronavirus disease (COVID-19) outbreak. J. Autoimmun..

[24] Mani J.S., Johnson J.B., Steel J.C., Broszczak D.A., Neilsen P.M., Walsh K.B., Naiker M. (2020). Natural product-derived phytochemicals as potential agents against coronaviruses: A review. Virus Res..

[25] Li F. (2016). Structure, function, and evolution of coronavirus spike proteins. Annu. Rev. Virol..

[26] Belen-Apak F.B., Sarialioglu F. (2020). The old but new: Can unfractioned heparin and low molecular weight heparins inhibit proteolytic activation and cellular internalization of SARS-CoV2 by inhibition of host cell proteases?. Med. Hypotheses.

[27] Daniloski Z., Jordan T.X., Ilmain J.K., Guo X., Bhabha G., Sanjana N.E. (2020). The Spike D614G mutation increases SARS-CoV-2 infection of multiple human cell types. bioRxiv.

[28] Zhang L., Jackson C.B., Mou H., Ojha A., Rangarajan E.S., Izard T., Farzan M., Choe M. (2020). The D614G mutation in the SARS-CoV-2 spike protein reduces S1 shedding and increases infectivity. bioRxiv.

[29] Plante J.A., Liu Y., Liu J., Xia H., Johnson B.A., Lokugamage K.G., Zhang X., Muruato A.E., Zou J., Fontes-Garfias C.R. (2020). Spike mutation D614G alters SARS-CoV-2 fitness. Nature.

[30] Li Q., Wu J., Nie J., Zhang L., Hao H., Liu S., Zhao C., Zhang Q., Liu H., nie L. (2020). The Impact of Mutations in SARS-CoV-2 Spike on Viral Infectivity and Antigenicity. Cell.

[31] Malik Y.A. (2020). Properties of Coronavirus and SARS-CoV-2. Malays J. Pathol..

[32] Hulswit R.J., de Haan C.A., Bosch B.J. (2016). Coronavirus Spike Protein and Tropism Changes. Adv. Virus Res..

[33] Wang Q., Zhang Y., Wu L., Niu S., Song C., Zhang Z., Lu G., Qiao C., Hu Y., Yuen K.Y. (2020). Structural and Functional Basis of SARS-CoV-2 Entry by Using Human ACE2. Cell.

[34] Romano M., Ruggiero A., Squeglia F., Maga G., Berisio R.A. (2020). Structural View of SARS-CoV-2 RNA Replication Machinery: RNA Synthesis, Proofreading and Final Capping. Cells.

[35] Chakraborty C., Sharma A.R., Sharma G., Bhattacharya M., Lee S.S. (2020). SARS-CoV-2 causing pneumonia-associated respiratory disorder (COVID-19): Diagnostic and proposed therapeutic options. Eur. Rev. Med. Pharmacol. Sci..

[36] Stadnytskyi V., Bax C.E., Bax A., Anfinrud P. (2020). The airborne lifetime of small speech droplets and their potential importance in SARS-CoV-2 transmission. Proc. Natl. Acad. Sci. USA.

[37] Lambert D.W., Clarke N.E., Turner A.J. (2010). Not just angiotensinases: New roles for the angiotensin-converting enzymes. Cell Mol. Life Sci..

[38] Perlot T., Penninger J.M. (2013). ACE2-from the renin-angiotensin system to gut microbiota and malnutrition. Microbes Infect..

[39] Gheblawi M., Wang K., Viveiros A., Nguyen Q., Zhong J.C., Turner A.J., Raizada M.K., Grant M.B., Oudit G.Y. (2020). Angiotensin-Converting Enzyme 2: SARS-CoV-2 Receptor and Regulator of the Renin-Angiotensin System: Celebrating the 20th Anniversary of the Discovery of ACE2. Circ. Res..

[40] Ocaranza M.P., Riquelme J.A., García L., Jalil J.E., Chiong M., Santos R.A.S., Lavandero S. (2020). Counter-regulatory renin–angiotensin system in cardiovascular disease. Nat. Rev. Cardiol..

[41] Roca-Ho H., Riera M., Palau V., Pascual J., Soler M.J. (2017). Characterization of ACE and ACE2 Expression within Different Organs of the NOD Mouse. Int. J. Mol. Sci..

[42] Nakamura K., Koibuchi N., Nishimatsu H., Higashikuni Y., Hirata Y., Kugiyama K., Nagai R., Sata M. (2008). Candesartan ameliorates cardiac dysfunction observed in angiotensin-converting enzyme 2-deficient mice. Hypertens. Res..

[43] Huang L., Sexton D.J., Skogerson K., Devlin M., Smith R., Sanyal I., Parry T., Kent R., Enright J., Wu Q.-L. (2003). Novel peptide inhibitors of angiotensin-converting enzyme 2. J. Biol. Chem..

[44] Corvol P., Williams T.A., Soubrier F. (1995). Peptidyl dipeptidase A: Angiotensin I-converting enzyme. Methods Enzymol..

[45] Donoghue M., Hsieh F., Baronas E., Godbout K., Gosselin M., Stagliano N., Donovan M., Woolf B., Robison K., Jeyaseelan R. (2000). A novel angiotensin-converting enzyme-related carboxypeptidase (ACE2) converts angiotensin I to angiotensin 1-9. Circ. Res..

[46] Tipnis S.R., Hooper N.M., Hyde R., Karran E., Christie G., Turner A.J. (2000). A human homolog of angiotensin-converting enzyme. Cloning and functional expression as a captopril-insensitive carboxypeptidase. J. Biol. Chem..

[47] Hikmet F., Méar L., Edvinsson A., Micke P., Uhlén M., Lindskog C. (2020). The protein expression profile of ACE2 in human tissues. Mol. Syst. Biol..

[48] Labò N., Ohnuki H., Tosato G. (2020). Vasculopathy and Coagulopathy Associated with SARS-CoV-2 Infection. Cells.

[49] Kuba K., Imai Y., Ohto-Nakanishi T., Penninger J.M. (2010). Trilogy of ACE2: A peptidase in the renin–angiotensin system, a SARS receptor, and a partner for amino acid transporters. Pharmacol. Ther..

[50] Vickers C., Hales P., Kaushik V., Dick L., Gavin J., Tang J., Godbout K., Parsons T., Baronas E., Hsieh F. (2002). Hydrolysis of Biological Peptides by Human Angiotensin-converting Enzyme-related Carboxypeptidase. J. Biol. Chem..

[51] Nadarajah R., Milagres R., Dilauro M., Gutsol A., Xiao F., Zimpelmann J., Kennedy C., Wysocki J., Batle D., Burns K.D. (2012). Podocyte-specific overexpression of human angiotensin-converting enzyme 2 attenuates diabetic nephropathy in mice. Kidney Int..

[52] Vuille-Dit-Bille R.N., Camargo S.M., Emmenegger L., Sasse T., Kummer E., Jando J., Hamie Q.M., Meier C.F., Hunziker S., Forras-Kaufmann Z. (2015). Human intestine luminal ACE2 and amino acid transporter expression increased by ACE-inhibitors. Amino Acids.

[53] Camargo S.M., Singer D., Makrides V., Huggel K., Pos K.M., Wagner C.A., Kuba K., Danilczyk U., Skovby F., Kleta R. (2009). Tissue-specific amino acid transporter partners ACE2 and collectrin differentially interact with hartnup mutations. Gastroenterology.

[54] Hashimoto T., Perlot T., Rehman A., Trichereau J., Ishiguro H. (2012). ACE2 links amino acid malnutrition to microbial ecology and intestinal inflammation. Nature.

[55] Arthur S., Singh S., Sundaram U. (2018). Cyclooxygenase pathway mediates the inhibition of Na-glutamine co-transporter B^0^AT1 in rabbit villus cells during chronic intestinal inflammation. PLoS ONE.

[56] Li F., Li W., Farzan M., Harrison S.C. (2005). Structure of SARS coronavirus spike receptor-binding domain complexed with receptor. Science.

[57] Kuba K., Imai Y., Rao S., Gao H., Guo F., Guan B., Huan Y., Yang P., Zhang Y., Deng W. (2005). A crucial role of angiotensin converting enzyme 2 (ACE2) in SARS coronavirus-induced lung injury. Nat. Med..

[58] Inoue Y., Tanaka N., Tanaka Y., Inoue S., Morita K., Zhuang M., Hattori T., Sugamura K. (2007). Clathrin-Dependent Entry of Severe Acute Respiratory Syndrome Coronavirus into Target Cells Expressing ACE2 with the Cytoplasmic Tail Deleted. J. Virol..

[59] Haga S., Yamamoto N., Nakai-Murakami C., Osawa Y., Tokunaga K., Sata T., Yamamoto N., Sasazuki T., Ishizaka Y. (2008). Modulation of TNF-alpha-converting enzyme by the spike protein of SARS-CoV and ACE2 induces TNF-alpha production and facilitates viral entry. Proc. Natl. Acad. Sci. USA.

[60] Wang H., Yang P., Liu K., Guo F., Zhang Y., Zhang G., Jiang C. (2008). SARS coronavirus entry into host cells through a novel clathrin- and caveolaeindependent endocytic pathway. Cell Res..

[61] Perrotta F., Matera M.G., Cazzola M., Bianco A. (2020). Severe respiratory SARS-CoV2 infection: Does ACE2 receptor matter?. Respir. Med..

[62] Qing E., Hantak M., Perlman S., Gallagher T. (2020). Distinct Roles for Sialoside and Protein Receptors in Coronavirus Infection. mBio.

[63] Nguyen H.L., Lan P.D., Thai N.Q., Nissley D.A., O’Brien E.P., Li M.S. (2020). Does SARS-CoV-2 Bind to Human ACE2 More Strongly Than Does SARS-CoV?. J. Phys. Chem. B.

[64] Santos R.A., Ferreira A.J., Verano-Braga T., Bader M. (2013). Angiotensin-converting enzyme2, angiotensin-(1–7) and Mas: New players of the renin-angiotensin system. J. Endocrinol..

[65] Qaradakhi T., Gadanec L.K., McSweeney K.R., Tacey A., Apostolopoulos V., Levinger I., Rimarova K., Egom E.E., Rodrigo L., Kruzliak P. (2020). The potential actions of angiotensin-converting enzyme II (ACE2) activator diminazene aceturate (DIZE) in various diseases. Clin. Exp. Pharmacol. Physiol..

[66] Kumar A., Narayan R.K., Kumari C., Faiq M., Kulandhasamy M., Kant K., Pareek V. (2020). SARS-CoV-2 cell entry receptor ACE2 mediated endothelial dysfunction leads to vascular thrombosis in COVID-19 patients. Med Hypotheses.

[67] Verdecchia P., Cavallini C., Spanevello A., Angeli F. (2020). The pivotal link between ACE2 deficiency and SARS-CoV-2 infection. Eur. J. Intern. Med..

[68] Teuwen L.A., Geldhof V., Pasut A., Carmeliet P. (2020). COVID-19: The vasculature unleashed. Nat. Rev. Immunol..

[69] Pagliaro P., Penna C. (2020). ACE/ACE2 Ratio: A Key Also in 2019 Coronavirus Disease (Covid-19)?. Front. Med..

[70] Thomas M.C., Pickering R.J., Tsorotes D., Koitka A., Sheehy K., Bernardi S., Toffoli B., Nguyen-Huu T.P., Head G.A., Fu Y. (2010). Genetic Ace2 deficiency accentuates vascular inflammation and atherosclerosis in the ApoE. Circ. Res..

[71] Chen I.Y., Chang S.C., Wu H.Y., Yu T.C., Wei W.C., Lin S., Chien C.L., Chang M.F. (2010). Upregulation of the chemokine (CC motif) ligand 2 via a severe acute respiratory syndrome coronavirus spike-ACE2 signaling pathway. J. Virol..

[72] Fraga-Silva R.A., Sorg B.S., Wankhede M., Dedeugd C., Jun J.Y., Baker M.B., Li Y., Castellano R.K., Katovich M.J., Raizada M.K. (2010). ACE2 activation promotes antithrombotic activity. Mol. Med..

[73] Hippisley-Cox J., Young D., Coupland C., Channon K.M., Tan P.S., Harrison D.A., Rowan K., Aveyard P., Pavord I.D., Watkinson P.J. (2020). Risk of severe COVID-19 disease with ACE inhibitors and angiotensin receptor blockers: Cohort study including 8.3 million people. Heart.

[74] Kiseleva R.Y., Glassman P.M., Greineder C.F., Hood E.D., Shuvaev V.V., Muzykantov V.R. (2018). Targeting therapeutics to endothelium: Are we there yet?. Drug Deliv. Transl. Res..

[75] Maiuolo J., Gliozzi M., Musolino V., Scicchiatano M., Carresi C., Scarano F., Bosco F., Nucera S., Ruga S., Zito M.C. (2018). The “Frail” Brain Blood Barrier in Neurodegenerative Diseases: Role of Early Disruption of Endothelial Cell-to-Cell Connections. Int. J. Mol. Sci..

[76] Maiuolo J., Gliozzi M., Musolino V., Carresi C., Nucera S., Macrì R., Scicchitano M., Bosco F., Scarano F., Ruga S. (2019). The Role of Endothelial Dysfunction in Peripheral Blood Nerve Barrier: Molecular Mechanisms and Pathophysiological Implications. Int. J. Mol. Sci..

[77] Keaney J., Campbell M. (2015). The dynamic blood-brain barrier. FEBS J..

[78] Durak-Kozica M., Baster Z., Kubat K., Stępień E. (2018). 3D visualization of extracellular vesicle uptake by endothelial cells. Cell Mol. Biol. Lett..

[79] Radeva M.Y., Waschke J. (2018). Mind the gap: Mechanisms regulating the endothelial barrier. Acta Physiol..

[80] Mundi S., Massaro M., Scoditti E., Carluccio M.A., Van Hinsbergh V.W.M., Iruela-Arispe M.L., De Caterina R. (2018). Endothelial permeability, LDL deposition, and cardiovascular risk factors—A review. Cardiovasc. Res..

[81] Short K.R., Kasper J., van der Aa S., Andeweg A.C., Zaaraoui-Boutahar F., Goeijenbier M., Richard M., Herold S., Becker C., Scott D. (2016). Influenza virus damages the alveolar barrier by disrupting epithelial cell tight junctions. Eur. Respir. J..

[82] Armstrong S.M., Darwish I., Lee W.L. (2013). Endothelial activation and dysfunction in the pathogenesis of influenza A virus infection. Virulence.

[83] Wang J., Li Q., Yin Y., Zhang Y., Cao Y., Lin X., Huang L., Hoffmann D., Lu M., Qiu Y. (2020). Excessive Neutrophils and Neutrophil Extracellular Traps in COVID-19. Front. Immunol..

[84] Green C.E., Turner A.M. (2017). The role of the endothelium in asthma and chronic obstructive pulmonary disease (COPD). Green Turn. Respir. Res..

[85] Janga H., Cassidy L., Wang F., Spengler D., Oestern-Fitschen S., Krause M.F., Seekamp A., Tholey A., Fuchs S. (2018). Site-specific and endothelial-mediated dysfunction of the alveolar-capillary barrier in response to lipopolysaccharides. J. Cell. Mol. Med..

[86] Tuder R.M., Yoshida T. (2011). Stress Responses Affecting Homeostasis of the Alveolar Capillary Unit. Proc. Am. Thorac. Soc..

[87] Kaur U., Acharya K., Mondal R., Singh A., Saso L., Chakrabarti S., Chakrabarti S.S. (2020). Should ACE2 be given a chance in COVID-19 therapeutics: A semi-systematic review of strategies enhancing ACE2. Eur. J. Pharmacol..

[88] Varga V., Flammer A.J., Steiger P., Haberecker M., Andermatt R., Zinkernagel A.S., Mehra M.R., Schueqbach R.A., Ruschitzka E., Moch H. (2020). Endothelial cell infection and endotheliitis in COVID-19. Lancet.

[89] Opal S.M., van der Poll T. (2015). Endothelial barrier dysfunction in septic shock. J. Intern. Med..

[90] Zhang J., Tecson K.M., McCullough P.A. (2020). Endothelial dysfunction contributes to COVID-19-associated vascular inflammation and coagulopathy. Rev. Cardiovasc. Med..

[91] Goshua G., Pine A.B., Meizlish M.L., Chang C.-H., Zhang H., Bahel P., Baluha A., Bar N., Bona R.D., Burns A.J. (2020). Endotheliopathy in COVID-19-associated coagulopathy: Evidence from a single-centre, cross-sectional study. Lancet Haematol..

[92] Ladikou E.E., Sivaloganathan A.H., Kate A., Milne M., William A., Ramasamy R., Saad R., Stoneman S.M., Eziefula A.C., Cheevassut T. (2020). Von Willebrand factor (vWF): Marker of endothelial damage and thrombotic risk in COVID-19?. Clin. Med..

[93] Menter T., Haslbauer J.D., Nienhold R., Savic S., Hopfer H., Deigendesch N., Frank S., Turek D., Willi. N., Pargger H. (2020). Post-mortem examination of COVID19 patients reveals diffuse alveolar damage with severe capillary congestion and variegated findings of lungs and other organs suggesting vascular dysfunction. Histopathology.

[94] Su H., Yang M., Wan C., Yi L.-X., Tang F., Zhu H.-Y., Yi F., Yang H.-C., Fogo A.B., Nie X. (2020). Renal histopathological analysis of 26 postmortem findings of patients with COVID-19 in China. Kidney Int..

[95] Carlson J.A., Cavaliere L.F., Grant-Kels J.M. (2006). Cutaneous vasculitis: Diagnosis and management. Clin. Dermatol..

[96] Freeman E.E., McMahon D.E., Fitzgerald M.E., Fox L.P., Rosenbach M., Takeshita J., French L.E., Thiers B.H., Hruza G.J. (2020). The American Academy of Dermatology COVID-19 registry: Crowdsourcing dermatology in the age of COVID-19. J. Am. Acad. Dermatol..

[97] Joob B., Wiwanitkit V. (2020). COVID-19 can present with a rash and be mistaken for Dengue. J. Am. Acad. Dermatol..

[98] Recalcati S. (2020). Cutaneous manifestations in COVID-19: A first perspective. JEADV.

[99] Mazzotta F., Troccoli T., Bonifazi E. (2020). Acute acro-ischemia in the child at the time of covid-19. Eur. J. Pediatric Dermatol..

[100] Criado P.R., Martinez Zugaib Abdalla B., Carvalho de Assis I., van Blarcum de Graaff Mello C., Cacciolari Caputo G., Campos Vieira I. (2020). Are the cutaneous manifestations during or due to SARS-CoV-2 infection/COVID-19 frequent or not? Revision of possible pathophysiologic mechanisms. Inflamm. Res..

[101] Castelnovo L., Capelli F., Tamburello A., Faggioli P.M., Mazzone A. (2020). Symmetric cutaneous vasculitis in COVID-19 pneumonia. JEADV.

[102] Suganya N., Bhakkiyalakshmi E., Sarada D.V.L., Ramkumar K.M.R. (2016). Reversibility of endothelial dysfunction in diabetes: Role of polyphenols. Br. J. Nutr..

[103] Mollace V., Tavernese A., Mollace R. (2020). The essential role of a “healthy” relationship between caveolin-1 and endothelial nitric oxide synthase in counteracting vascular inflammation and atherothrombosis. Kardiol. Pol..

[104] Kovacs-Kasa A., Kim K.M., Cherian-Shaw M., Black S.M., Fulton D.J., Verin A.D. (2018). Extracellular adenosine-induced Rac1 activation in pulmonary endothelium: Molecular mechanisms and barrier-protective role. J. Cell Physiol..

[105] Ohmura T., Tian Y., Sarich N., Ke Y., Meliton A., Shah A.S., Andreasson K., Birukov K.G., Birukova A.A. (2017). Regulation of lung endothelial permeability and inflammatory responses by prostaglandin A2: Role of EP4 receptor. Mol. Biol. Cell..

[106] Zhang J., Lu X., Liu M., Fan H., Zheng H., Zhang S., Rahman N., Wołczyński S., Kretowski A., Li X. (2019). Melatonin inhibits inflammasome-associated activation of endothelium and macrophages attenuating pulmonary arterial hypertension. Cardiovasc. Res..

[107] Konya V., Üllen A., Kampitsch N., Theiler A., Philipose S., Parzmair G.P., Marsche G., Peskar B.A., Schuligoi R., Sattler W. (2013). Endothelial E-type prostanoid 4 receptors promote barrier function and inhibit neutrophil trafficking. J. Allergy Clin. Immunol..

[108] Karki P., Birukov K.G. (2020). Oxidized Phospholipids in Healthy and Diseased Lung Endothelium. Cells.

[109] Fu P., Shaaya M., Harijith A., Jacobson J.R., Karginov A., Natarajan V. (2018). Sphingolipids Signaling in Lamellipodia Formation and Enhancement of Endothelial Barrier Function. Curr. Top. Membr..

[110] Zheng B., Ye L., Zhouguang W., Zhu S., Wang Q., Shi H., Chen D., Wei X., Wang Z., Li X. (2016). Epidermal growth factor attenuates blood-spinal cord barrier disruption via PI 3K/Akt/Rac1 pathway after acute spinal cord injury. J. Cell. Mol. Med..

[111] Oppedisano F., Maiuolo J., Gliozzi M., Musolino V., Carresi C., Nucera S., Scicchitano M., Scarano F., Bosco F., Macrì R. (2020). The Potential for Natural Antioxidant Supplementation in the Early Stages of Neurodegenerative Disorders. Int. J. Mol. Sci..

[112] Gliozzi M., Scicchitano M., Bosco F., Musolino V., Carresi C., Scarano F., Maiuolo J., Nucera S., Maretta A., Paone A. (2019). Modulation of Nitric Oxide Synthases by Oxidized LDLs: Role in Vascular Inflammation and Atherosclerosis Development. Int. J. Mol. Sci..

[113] Xin Z., Guan X., Zhi B.Z. (2014). Resveratrol Attenuates Hypoxia-Reperfusion Injury Induced Rat Myocardium Microvascular Endothelial Cell Dysfunction Through Upregulating PI3K/Akt/SVV Pathways. Zhonghua Xin Xue Guan Bing Za Zhi.

[114] Mu S., Liu Y., Jiang J., Ding R., Li X., Li X., Ma X. (2018). Unfractionated heparin ameliorates pulmonary microvascular endothelial barrier dysfunction via microtubule stabilization in acute lung injury. Respir. Res..

[115] Slobodnick A., Shah B., Pillinger M.H., Krasnokutsky S. (2015). Colchicine: Old and new. Am. J. Med..

[116] Cure C., Kucuk A., Cure E. Colchicine may not be effective in COVID-19 infection; it may even be harmful?. Clin. Rheumatol..

[117] Kollias A., Kyriakoulis K.G., Dimakakos E., Poulakou G., Stergiou G.S., Syrigos K. (2020). Thromboembolic risk and anticoagulant therapy in COVID-19 patients: Emerging evidence and call for action. Br. J. Haematol..

[118] Poterucha T.J., Libby P., Goldhaber S.Z. (2017). More than an anticoagulant: Do heparins have direct anti-inflammatory effects?. Thromb. Haemost..

[119] Thachil J. (2020). The versatile heparin in COVID-19. Int. Soc. Thromb. Haemost..

[120] Chen N., Zhou M., Dong X., Qu J., Gong F., Han Y., Qiu Y., Wang J., Liu Y., Wei Y. (2020). Epidemiological and clinical characteristics of 99 cases of 2019 novel coronavirus pneumonia in Wuhan, China: A descriptive study. Lancet.

[121] Delabranche X., Helms J., Meziani F. (2017). Immunohaemostasis: A new view on haemostasis during sepsis. Ann. Intensive Care.

[122] Jackson S.P., Darbousset R., Schoenwaelder S.M. (2019). Thromboinflammation: Challenges of therapeutically targeting coagulation and other host defense mechanisms. Blood.

[123] Huertas A., Montani D., Savale L., Pichon J., Tu L., Parent F., Guignabert C., Humbert M. (2020). Endothelial cell dysfunction: A major player in SARS-CoV-2 infection (COVID-19)?. Eur. Respir. J..

